# Diabetes and prediabetes prevalence among young and middle-aged adults in India, with an analysis of geographic differences: findings from the National Family Health Survey

**DOI:** 10.4178/epih.e2020065

**Published:** 2020-09-18

**Authors:** Siddardha G. Chandrupatla, Isma Khalid, Tejdeep Muthuluri, Satyanarayana Dantala, Mary Tavares

**Affiliations:** 1 Department of Oral Health Policy and Epidemiology, Harvard School of Dental Medicine, Boston, MA, USA; 2 Department of Pediatric Dentistry, Boston University, Boston, MA, USA; 3 Deptartment of Oral Surgery, SVS Dental College, Mehboobnagar, India; 4 NTR University of Health Sciences, Panineeya Dental College, Hyderabad, India

**Keywords:** Diabetes prevalence, Body mass index, Obesity Demographic and Health Survey, India

## Abstract

**OBJECTIVES:**

This study aimed to approximate the prevalence of hyperglycemia in India.

**METHODS:**

The study was conducted using the Demographic and Health Survey 2015-16 (also known as the National Family Health Survey-4), which surveyed 811,808 individuals using a stratified, multistage, cluster sampling design. This cross-sectional survey recorded socio-demographic and anthropometric data, including blood glucose levels, of adults aged 18 years to 54 years.

**RESULTS:**

The final analysis included 718,597 individuals, of whom 49.90% (weighted) were males. The overall prevalence of diabetes was 6.65% and that of prediabetes was 5.57%. A positive association was seen with urban residence, geographic region, sex, age, body mass index, socioeconomic status, and hypertension. Approximately two-thirds of individuals with diabetes lived in urban areas, and about half of the urban population was considered overweight/obese. South India showed a higher prevalence of diabetes (prevalence ratio, 2.01; p<0.001) than northern India.

**CONCLUSIONS:**

Hyperglycemia (diabetes and prediabetes) has a high prevalence in India and is a major public health issue. Diabetes is unevenly distributed based on geographic location and urbanization. Prevention, early detection, and treatment strategies should consider this uneven distribution of diabetes.

## INTRODUCTION

India has the second-largest population in the world and may surpass China as the world’s most populous nation by 2024. Nearly 32% of Indians live in urban areas (2011 census) [1], an increase from 27% in 2001. Increasingly many people are employed in white-collar jobs that require less physical activity. In relation to these changes, type 2 diabetes is a disease that has a multifactorial etiology including genetic and lifestyle factors [2,3]. Similar to major metropolitan areas across the world, Indian cities display a higher prevalence of type 2 diabetes than rural areas [4]. With the increase in people living a more sedentary lifestyle, paralleled with diets high in carbohydrates and fat, there has been an increase in the number of cases of diabetes and related diseases across India [5].

Diabetes is rapidly becoming an epidemic in India, with more than 74 million individuals diagnosed with the disease [6]. In 2017, approximately 425 million people worldwide were recognized as having diabetes, and this number is likely to reach 629 million by 2045 [6]. Each year, approximately 1.7 million deaths occur due to type 2 diabetes, making it one of the leading causes of mortality. India currently faces an uncertain future in relation to the potential burden that diabetes may impose upon the country. It is predicted that by 2030, 80 million to 100 million individuals in India may have type 2 diabetes [5].

The purpose of this study was to approximate the prevalence of type 2 diabetes among adults in India and to compare differences in prevalence based on socioeconomic factors, including wealth and geographic factors. This is the first survey to present data on biomarkers sampled from across India.

## MATERIALS AND METHODS

Data were collected from the Demographic and Health Surveys-VII (DHS-VII) of India, also known as the National Family Health Survey-4 (NFHS-4). The NFHS is a survey of nationally representative households that provides data for the monitoring and evaluation of population, health, and nutrition in India. The NFHS-4 was conducted in 18 languages from 2015 to 2016, among 601,509 households (811,808 subjects), including 699,686 female respondents and 112,122 male respondents. The response rate was 97.6% for the household interviews, 96.7% for the females interviewed, 91.9% for the males interviewed, and 95.6% for blood glucose tests [7]. Of these surveys, 757,958 were complete, including biomarker sampling. For the final analysis, we excluded females who were pregnant at the time of the survey (4.83%) and those with incomplete data (1.72% with incomplete blood glucose data). The DHS surveys are designed to collect data on marriage, fertility, family planning, reproductive health, child health, and HIV/AIDS. To achieve the survey objectives, females (15-49 years) of reproductive age are main focus of the survey. Due to the focus on female subjects, fewer males were surveyed. Sufficient sampling weights were attached to each male participant to represent the entire population.

Anthropometric measurements, hemoglobin levels, blood pressure (BP), and blood glucose levels were collected from females aged 15-49 years and males aged 15-54 years. For this analysis, the Individual Recode dataset and Men’s Recode datasets were appended and then merged with the Household Recode dataset. The NFHS-4 is the first DHS survey in India to incorporate blood glucose levels, human immunodeficiency virus testing, and BP readings recorded at the district level. Blood glucose levels were measured in males and females aged 18 years to 54 years who provided samples for measurement. Blood glucose was tested using the Freestyle Optium H glucometer (Abbott Diabetes Care Inc., Alameda, CA, USA).

### Outcome variable

The outcome variable was the presence of high serum blood glucose levels. The diagnosis of diabetes was made based on the values of serum glucose levels as prescribed by the World Health Organization (WHO) in 2019 [8,9]. The participants were divided into 2 categories based on the time interval between food intake and the collection of blood samples. For participants who did not consume any food or drinks (other than water) for the past 8 hours, a blood glucose level of > 126 mg/dL was used as the cut-off for diagnosing diabetes (fasting glucose level) and a value of 100 mg/dL to 125 mg/dL was considered to indicate prediabetes. For those who had eaten or had drunk liquids other than water within the past 8 hours, a blood glucose level of > 200 mg/dL was considered to indicate diabetes (random blood glucose levels) and levels from 140 mg/dL to 199 mg/dL were considered to indicate prediabetes [9,10]. Participants who answered “yes” to a question asking them if they had been diagnosed with diabetes were categorized as having diabetes.

### Predictor variables

The main predictor variables included geographic location and place of residence. The geographic location was categorized as (1) northern, (2) eastern and northeast, (3) western, (4) central, and (5) southern India. Place of residence was dichotomized as (1) urban and (2) rural. Other predictor variables included the age of the participant, sex, educational attainment, wealth index, type of employment, presence of hypertension, body mass index (BMI), and the daily consumption of carbonated drinks. The wealth index was derived through principal component analysis from an index of standard household assets and indicators of housing quality. Based on this the participants were categorized into 5 categories: (1) poorest, (2) poor, (3) middle, (4) rich, and (5) richest. For our analysis, the poor and poorest categories were combined. Participants’ educational attainment was categorized as no education (0 years of education), primary (1-5 years of education), secondary (6-8 years of education), and high school and above (≥9 years of education). The type of employment was categorized as low-physical-demands and high-physical-demands/labor-intense jobs. Participants who were currently working in a professional, managerial, clerical, or sales job were categorized as having low-physical-demands jobs. Agriculture, domestic services, and manual jobs were grouped as high-physical-demands jobs. BMI was classified based on the WHO cut-off values for Asian populations as normal weight (18.5-22.9 kg/m^2^), overweight (23.0-24.9 kg/m^2^), and obese (≥25.0 kg/m^2^). The consumption of carbonated drinks was categorized as daily or non-daily consumption. In the NFHS-4, the BP was taken and recorded 3 times for each participant, with 10-minute intervals between each recording. For the analysis, the average of the second and third BP readings was considered. Hypertension was defined as a systolic BP of ≥140 mmHg, a diastolic BP of ≥90 mmHg, or a current treatment plan with antihypertensive medication.

### Statistical analysis

A descriptive analysis was performed for all variables based on the presence of diabetes. All the categorical variables were tested using the chi-square test. The survey data were scaled for the presence of any stratum with a single sampling unit. Logistic regression was performed to calculate the unadjusted and adjusted odds ratios for the presence of type 2 diabetes. The regression analysis was done by testing the interaction terms of important variables associated with diabetes/hyperglycemia. Variables that were not significant were not used in the final regression model. All analyses were conducted using Stata version 14 (StataCorp., College Station, TX, USA).

### Ethics statement

The de-identified data was obtained after a written permission from the DHS.

## RESULTS

Table 1 presents the demographic characteristics of the population and their diabetes status. A total of 718,597 participants were included in the final analysis, of whom 6.65% had diabetes and 5.57% had prediabetes. Of those participants, about 619,833 were female and 98,764 were male (49.90% weighted). The mean age of the sample was 31.7 years (data not shown). In the age group of 35-49 years, 6.63% of participants had prediabetes and 9.19% had diabetes. Of the males aged 50-54 years, 15.22% had diabetes and 7.18% had prediabetes. Most of the participants (48.0%) had at least a secondary education, while 16.6% had a higher education and about 22.0% did not have any education. Those employed in high-physical-demands occupations or manual labor had lower prevalence (6.87%) of diabetes than those with professional or low-physical-demands jobs (10.11%). Among the wealth index groups, the poor/poorest had the lowest prevalence of diabetes (4.35%), the middle class had a prevalence of 5.91%, the richer class had a prevalence of 7.76%, and the richest class had a 9.79% prevalence of diabetes. In contrast, prediabetes was less common among the wealthier classes (4.93 and 5.48%) than among the poor (6.06%). The highest prevalence of diabetes was found in south India (9.39%), followed by eastern (6.81%) and western Indian (6.58%). Northern India had the lowest prevalence of diabetes (4.90%) and prediabetes (4.86%). Approximately 12.6% of adults had hypertension and the prevalence of diabetes and prediabetes were higher among people with hypertension. A higher proportion (10.19%) of adults with hypertension had diabetes than adults without hypertension (6.14%). Prediabetes was observed in 6.75% of people with hypertension, compared to 5.40% of people without hypertension. The prevalence of diabetes and prediabetes increased with BMI. Obese people had a higher prevalence of diabetes (9.85%) and prediabetes (6.75%) than people in other categories.

Table 2 presents data for the subjects who had been diagnosed with diabetes and hypertension by their healthcare providers. Approximately 2.1% of the sample had previously been diagnosed with diabetes by their healthcare provider. Of these, 78.0% were currently on diabetes control medication. Table 3 shows the distribution of some key factors for diabetes in urban and rural areas. These factors were educational attainment, BMI, occupation, health insurance, and hypertension. Approximately half of the total population (48.1%) had secondary education, followed by 21.9% who did not have any education. Among those with higher education, the majority resided in urban areas (58.8 vs. 41.2%). Approximately 45.8% of participants could be categorized as overweight (or obese) by BMI. Among those with a normal BMI, 32.2% lived in urban areas and 67.8% lived in rural areas. About 12.4% of the entire population had elevated blood pressure readings. Among those with hypertension, 39.9% lived in urban areas and 60.1% lived in rural areas.

Table 4 presents the unadjusted and adjusted multivariable logistic regression results for the presence of type 2 diabetes. Older age, male sex, urban residence, being from eastern and southern India, a higher socioeconomic status (SES), and hypertension showed positive relationships with having diabetes. Males had 1.34 higher odds of having diabetes than females (p<0.001). The richer and richest SES groups had 1.83 and 2.38 higher odds of having diabetes compared to the poor/poorest groups respectively (p<0.001). Compared to north Indians, people residing in south India had 2.01 higher unadjusted odds of having diabetes (p<0.001). In the region of central India, residents showed statistically significantly higher odds of having diabetes than residents of north India when adjusting for other risk factors. Obese participants had 2.71 higher odds of having diabetes and overweight individuals had 1.57 higher odds. People with hypertension were 1.73 times more likely to have diabetes than those who did not (p<0.001).

## DISCUSSION

The previous NFHS survey in India was conducted in 2005, so the data from the latest survey shed important new light on the current health status. This is the first NFHS dataset where blood samples were collected for analysis. We estimated the prevalence of diabetes and the geographical differences in its prevalence within a nationally representative sample in India. The prevalence of diabetes and prediabetes varied significantly based on the geographic location, place of residence, SES, the presence of hypertension, and BMI.

In our study, 6.65% of the population surveyed had diabetes and 5.57% had prediabetes. Although numerous studies have been conducted in India, only a few multicenter studies have aimed to estimate the prevalence of diabetes in India. The Prevalence of Diabetes in India Study reported a diabetes prevalence of 4.3% in India, with rates of 5.9% in urban areas and 2.7% in rural areas [11]. This may be the only other study that was conducted throughout India and was based on a nationally representative sample. An earlier multicenter study in India reported a diabetes prevalence of 2.1% in urban and 1.5% in rural areas [12]. The National Urban Diabetes Survey by Ramachandran et al.[13] reported prevalence rates of 12% for diabetes and 14% for impaired glucose tolerance in urban India. A large nationally representative study (Indian Council of Medical Research–INdia DIABetes) showed an overall prevalence of 7.2% for diabetes and 10.3% for prediabetes [14]. Another study based on a self-reporting survey found that the prevalence of diabetes ranged from 3.1% (rural) to 7.3% (urban) [15]. The findings of the present study on SES and diabetes prevalence are similar to those of many other studies, in that poorer segments of the population had lower rates of diabetes prevalence than wealthier segments. However, it is noteworthy that the prediabetes prevalence was lower among the rich (4.93%) and richer (5.48%) groups than in the poor group (6.06%; p<0.001). This finding is similar to the results of a study in Bangladesh, where the poor had a higher prevalence of prediabetes (23.7%) than the rich (19.7%) [10]. This finding of a higher prediabetes prevalence in poorer individuals should be further investigated. A possible reason may be that poorer individuals tend to engage in higher levels of daily physical activities at the workplace, which may prevent the progression to diabetes.

The findings of our study are similar to those of many other studies regarding the higher diabetes prevalence in urban locations. The higher prevalence of diabetes among urban residents has been reported in many studies, including those conducted in Asian countries, such as China, Bangladesh, and Iran [10,16-18]. A study based in China showed that the prevalence of diabetes was higher among urban residents than among rural residents (11.4 vs. 8.2%) [16]. Bangladesh, which is a neighbor of India and was formerly part of British India, is highly similar to eastern Indian states in terms of population, culture, and diet. Hussain et al. [19] reported a higher prevalence of diabetes among urban residents (8.1%) than among rural residents (2.3%). Another study in Bangladesh showed that the prevalence of diabetes was 9.7% and that of prediabetes was 22.4%. Among urban residents, the prevalence of diabetes was 15.2%, while it was 8.3% among rural residents [10]. The higher prevalence of diabetes and prediabetes among urban residents may be attributed to their intake of a higher-calorie diet accompanied with lower physical activity [20,21].

Age, BMI, and hypertension also showed positive associations with diabetes prevalence, which is a similar finding to those of other studies [22,25]. In our study, prediabetes (8.1%) and diabetes (10.3%) showed a high prevalence among males aged 50 years to 54 years. Asians, including Indians, have a higher susceptibility to diabetes due to greater abdominal and visceral obesity at any given BMI [25,26]. The WHO BMI cut-off points for risk assessment of diabetes are therefore lower for Asian populations than for other races [27,28]. Using the WHO cut-off points for BMI, our study identified that 26.6% of adults were obese and 18.8% were overweight. Individuals who were overweight (odds ratio [OR], 1.57; p<0.001) and obese (OR, 2.71; p<0.001) had a higher risk of diabetes.

The main strength of our study is the large sample size. The study was conducted in all states of India and was designed to represent the Indian population at the district level. Many variables were recorded along with blood glucose measurements, which helped in assessing the associations of diabetes and prediabetes with other factors. However, the cross-sectional nature of the study does not allow for the establishment of causal relationships. The dietary questionnaire findings may be biased due to the dependence on the recall memory of the survey participants. The survey was conducted as part of the global DHS program, with a focus on female’s reproductive health (i.e., in females from 14 years to 59 years of age), and the survey therefore did not capture the classic symptoms of hyperglycemia, such as frequency of urination and excessive thirst. Instead, hyperglycemia was assessed by random measurements of glucose levels. This warrants further research into the prevalence of diabetes at the national level with inclusion of the symptoms of diabetes in DHS surveys across the world. Individuals who have elevated random blood glucose levels, regardless of whether they have the classic symptoms of diabetes or are asymptomatic, need to undergo repeat testing for a diagnosis. Therefore, this aspect of the study design may have led to an overestimation of the prevalence of diabetes.

India faces several challenges to tackle the diabetes epidemic. The increasing population of India is placing more pressure on the limited supply of healthcare professionals. Innovative healthcare solutions such as telemedicine, group visit models, and the use of allied healthcare professionals are urgently warranted in India to identify undiagnosed cases and to facilitate the early detection of diabetes [29-31].

## Figures and Tables

**Table 1.** Socioeconomic, demographic, and anthropometric characteristics of the study participants by diabetes status- National Family Health Survey-4, 2015-16

Characteristics	Normal	Pre-diabetes	Diabetes	p-value (chi-square)	Total (column)
Age (yr)				<0.001	
18-25	91.68	4.56	3.75		30.0
26-34	90.03	4.98	4.98		27.8
35-49	84.16	6.63	9.19		37.9
50-54^1^	77.59	7.18	15.22		4.3
Sex				<0.001	
Female	88.84	5.41	5.74		50.1
Male	86.67	5.74	7.57		49.9
Education				<0.001	
No education	87.59	6.44	5.96		22.0
Primary	87.20	5.79	7.01		13.3
Secondary	88.06	5.39	6.54		48.0
Higher	87.57	4.80	7.61		16.6
Employment type				<0.001	
Manual/high physical demands	87.22	5.89	6.87		77.1
Professional/low physical demands	83.90	5.97	10.11		22.9
Wealth index					
Poor/poorest	89.53	6.06	4.35	<0.001	34.7
Middle	88.51	5.57	5.91		20.8
Richer	86.75	5.48	7.76		21.9
Richest	85.26	4.93	9.79		22.6
Place of residence				<0.001	
Urban	85.36	5.39	9.24		36.7
Rural	89.15	5.68	5.15		63.3
Geographic location				<0.001	
North	90.22	4.86	4.90		27.3
East and northeast	86.88	6.30	6.81		23.8
West	88.45	4.96	6.58		16.4
Central	89.13	6.49	4.36		8.4
South	84.83	5.77	9.39		23.8
Hypertension				<0.001	
Yes	83.04	6.75	10.19		12.6
No	88.44	5.40	6.14		87.4
Body mass index				<0.001	
Normal	90.77	5.35	3.86		54.6
Overweight	88.73	5.30	5.95		18.8
Obese	83.38	6.75	9.85		26.6
Health insurance				<0.001	
None	88.52	5.37	6.09		70.5
Public	85.99	6.26	7.75		26.1
Private	85.99	4.60	9.39		3.4
Total	87.76	5.57	6.65		100

Values are presented as weighted %.

1 Males only.

**Table 2.** Current status of diabetes and hypertension

Variables	Female	Male	Total
Previosly diagnosed with diabetes	1.9	2.3	2.1
Currently seeking treatment for diabetes control among those diagnosed with diabetes	81.8	76.5	78.8
Participant was told that he or she had high blood pressure on 2 or more occasions by a doctor	10.0	15.1	8.8
Currently using medication for hypertension prescribed by a health provider	11.4	7.6	9.1

Values are presented as percentage.

**Table 3.** Distribution of education, body mass index, and hypertension by place of residence, India National Family Health Survey-4, 2015-2016

Variables	All	Urban	Rural	p-value
Education level				<0.001
No education	21.9	20.9	79.2	
Primary education	13.3	29.0	71.0	
Secondary education	48.1	38.8	61.2	
Higher education	16.7	58.8	41.2	
Body mass index				<0.001
Normal	54.2	32.2	67.8	
Overweight/obese	45.8	48.9	51.9	
Occupation type				<0.001
Professional/clerical/skilled	23.1	61.3	38.6	
Manual/labor	76.9	29.6	70.3	
Health insurance				0.097
No	70.4	37.2	62.8	
Yes	29.6	63.9	36.1	
Hypertension				<0.001
Yes	12.4	39.9	60.1	
No	87.6	36.5	63.5	

Values are presented as percentage.

**Table 4.** Estimates of ORs and 95% CIs of various correlates of diabetes, India National Family Health Survey-4, 2015-2016

Variables	OR (95% CI)	p-value	aOR (95% CI)	p-value
Age (yr)				
18-25	1.00 (reference)		1.00 (reference)	
26-34	1.34 (1.24, 1.44)	<0.001	1.31 (1.18, 1.44)	<0.001
35-49	2.59 (2.42, 2.77)	<0.001	2.83 (2.58, 3.10)	<0.001
50-54	4.59 (4.08, 5.17)	<0.001	4.74 (4.10, 5.49)	<0.001
Sex				
Female	1.00 (reference)		1.00 (reference)	
Male	1.34 (1.27, 1.42)	<0.001	1.14 (1.08, 1.21)	<0.001
Wealth index				
Poorest and poor	1.00 (reference)		1.00 (reference)	
Middle	1.38 (1.28, 1.48)	<0.001	1.16 (1.07, 1.27)	<0.001
Richer	1.83(1.71, 1.99)	<0.001	1.30 (1.19, 1.42)	<0.001
Richest	2.38(2.21, 2.57)	<0.001	1.46 (1.32, 1.60)	<0.001
Place of residence				
Urban	1.00 (reference)		1.00 (reference)	
Rural	0.53 (0.49, 0.56)	<0.001	0.88 (0.82, 0.95)	0.002
Location of residence				
North	1.00 (reference)		1.00 (reference)	
East and northeast	1.42 (1.28, 1.56)	<0.001	1.78 (1.63, 1.95)	<0.001
West	1.36 (1.23, 1.51)	<0.001	1.19 (1.07, 1.32)	0.001
Central	0.89 (0.80, 0.96)	0.008	1.26 (1.16, 1.38)	<0.001
South	2.01 (1.84, 2.19)	<0.001	1.95 (1.80, 2.11)	<0.001
Body mass index				
Normal	1.00 (reference)		1.00 (reference)	
Overweight	1.57 (1.45, 1.69)	<0.001	1.23 (1.14, 1.33)	<0.001
Obese	2.71 (2.55, 2.88)	<0.001	1.83 (1.72, 1.96)	<0.001
Hypertension				
No	1.00 (reference)		1.00 (reference)	
Yes	1.73 (1.62, 1.84)	<0.001	1.47 (1.37, 1.57)	<0.001
Consume carbonated drinks daily				
No	1.00 (reference)		1.00 (reference)	
Yes	1.17 (1.04, 1.32)	0.009	0.97 (0.85, 1.10)	0.670

OR, odds ratio; CI, confidence interval; aOR, adjusted odds ratio.
